# Androgen response element of the glycine N-methyltransferase gene is located in the coding region of its first exon

**DOI:** 10.1042/BSR20130030

**Published:** 2013-09-17

**Authors:** Cheng-Ming Lee, Chia-Hung Yen, Tsai-Yu Tzeng, Yu-Zen Huang, Kuan-Hsien Chou, Tai-Jay Chang, Yi-Ming Arthur Chen

**Affiliations:** *AIDS Prevention and Research Center, National Yang-Ming University, Taipei, Taiwan 11221; †Graduate Institute of Natural Products, College of Pharmacy, Kaohsiung Medical University, Kaohsiung, Taiwan 80708; ‡Center for Infectious Disease and Cancer Research, Kaohsiung Medical University, Kaohsiung, Taiwan 80708; §VYM Genome Research Center, National Yang-Ming University, Taipei, Taiwan 11221; ∥Department of Medical Research and Education, Taipei Veterans General Hospital, Taiwan 11221; ¶Institute of Biotechnology in Medicine, National Yang-Ming University, Taipei, Taiwan 11221; **Department of Microbiology, School of Medicine, Kaohsiung Medical University, Kaohsiung, Taiwan 80708

**Keywords:** androgen receptor, androgen response element, chromatin immunoprecipitation, electrophoretic mobility shift assay, GNMT, prostate cancer, Yin and Yang 1, AR, androgen receptor, ARE, androgen response element, ChIP, chromatin immunoprecipitation, CS, charcoal-stripped, *C*_T_, cycle threshold, EMSA, electrophoretic mobility shift assay, GAPDH, glyceraldehyde-3-phosphate dehydrogenase, GNMT, glycine N-methyltransferase, mTOR, mammalian target of rapamycin, NF-Y, nuclear factor-Y, PCa, prostate cancer, PI3K, phosphoinositide 3-kinase, PKB, protein kinase B (also called Akt), RGA, reporter gene assay, RT–PCR, reverse transcription–PCR, SAH, S-adenosylhomocysteine, SAM, S-adenosylmethionine, SV40, simian virus 40, TBP, TATA-box-binding protein, wt, wild-type, YY1, Yin and Yang 1

## Abstract

Androgen plays an important role in the pathogenesis of PCa (prostate cancer). Previously, we identified GNMT (glycine N-methyltransferase) as a tumour susceptibility gene and characterized its promoter region. Besides, its enzymatic product-sarcosine has been recognized as a marker for prognosis of PCa. The goals of this study were to determine whether *GNMT* is regulated by androgen and to map its AREs (androgen response elements). Real-time PCR analyses showed that R1881, a synthetic AR (androgen receptor) agonist induced GNMT expression in AR-positive LNCaP cells, but not in AR-negative DU145 cells. *In silico* prediction showed that there are four putative AREs in *GNMT*-ARE1, ARE2 and ARE3 are located in the intron 1 and ARE4 is in the intron 2. Consensus ARE motif deduced from published AREs was used to identify the fifth ARE-ARE5 in the coding region of exon 1. Luciferase reporter assay found that only ARE5 mediated the transcriptional activation of R1881. ARE3 overlaps with a YY1 [Yin and Yang 1 (motif (CaCCATGTT, +1118/+1126)] that was further confirmed by antibody supershift and ChIP (chromatin immunoprecipitation) assays. EMSA (electrophoretic mobility shift assay) and ChIP assay confirmed that AR interacts with ARE5 *in vitro* and *in vivo*. In summary, *GNMT* is an AR-targeted gene with its functional ARE located at +19/+33 of the first exon. These results are valuable for the study of the influence of androgen on the gene expression of GNMT especially in the pathogenesis of cancer.

## INTRODUCTION

PCa (prostate cancer) is the third most common cancer for men throughout the planet, and the most common among men in North America, Europe and some parts of Africa [1]. Besides age, race and family history, identified risk factors include androgen, diet, physical activity, sexual history, inflammation and obesity [2]. Androgen plays a crucial role in the pathogenesis of PCa [3,4]. After interacting with androgen, the activated AR (androgen receptor) is translocated into the nucleus where it binds to AREs (androgen response elements) present in different target genes [5]. AR plays a pivotal role in the initiation and progression of PCa, mainly through promoting the growth and proliferation of PCa cells [6–8].

GNMT (glycine N-methyltransferase) affects epigenetic modification by regulating the ratio of SAM (S-adenosylmethionine) to SAH (S-adenosylhomocysteine) in one-carbon metabolism pathway [9]. The human *GNMT* gene primarily expresses in the liver, pancreas and prostate [10]. Previously, we used immunohistochemical staining to demonstrate that *GNMT* expresses abundantly in the normal and hyperplasia tissues of prostate, while its expression was down-regulated in more than 80% of PC tissues [11]. In addition, loss of heterozygosity among different GNMT alleles (including INS/DEL and STRP1) has been observed in 36.4% of PCa [11]. Therefore we postulated that GNMT is a susceptibility gene for PCa.

It is worth noting that Sreekumar et al. reported that there were significant increases of the levels of sarcosine, the enzymatic reaction product of GNMT in invasive PCa cell lines compared with benign prostate epithelial cells [12]. Furthermore, they found that the invasiveness of PCa cells was attenuated if they knock down the *GNMT* gene expression [12]. It suggests that *GNMT* not only plays a role in the transformation and pathogenesis of PCa, but also is involved in the invasiveness and metastasis of PCa. Although we have characterized the promoter region and xenobiotic responsive elements of human *GNMT*, there is no report about the identification ARE so far [13]. Therefore the goals of this study were to test whether androgen can stimulate *GNMT* expression, and if yes, then we would like to further map its AREs. The results showed that *GNMT* is an androgen-inducing gene and a functional ARE is located in the coding region of the exon 1. It is intriguing to note that during our study, a YY1 (Ying and Yang 1) binding motif was accidently identified in the intron 1 of GNMT since it shares partial sequence homology with the ARE. These data have important implication to the future study of the interaction between hepatitis B viral infection and *GNMT* gene regulation.

## MATERIALS AND METHODS

### Cell cultures

A prostate adenocarcinoma cell line-LNCaP cells and its isogenic subline-C4-2 cells were cultured in RPMI 1640 (Gibco BRL) supplemented with 10% (v/v) FBS. PC3 cells, a prostate carcinoma cell line were cultured in Ham's F12K medium supplemented with 7% (v/v) FBS. The following three cell lines were cultured in DMEM (Dulbecco's modified Eagle's medium) supplemented with 10% FBS: DU145 (a prostate carcinoma cell line), HuH-7 [a HCC (hepatocellular carcinoma) cell line], and COS-1 (a green monkey kidney cell line). For the hormone-treatment experiment, we replaced FBS with CS (charcoal-stripped) FBS in steroid-depleted cell culture media.

### RT–PCR (reverse transcription–PCR) and real-time PCR

RT–PCR and real-time PCR were performed as described by Lee et al. previously [14]. The following primers were used in the real-time PCR: GNMT-F (5′-GCAGCCTTCGGAGGTAAGTG) and GNMT-R (5′-GGTTTGGCCTGGCTTGTAAG) for GNMT; ACTB-F (5′-GCCGGGACCTGACTGACTAC) and ACTB-R (5′-TCCTTAATGTCACGCACGATTT) for β-actin; TBP (TATA-box-binding protein)-F (5′-CAGAAGTTGGGTTTTCCAGCTAA) and TBP-R (5′-ACATCACAGCTCCCCACCAT) for TBP; and GAPDH (glyceraldehyde-3-phosphate dehydrogenase)-F (5′-TCACCACCATGGAGAAGGC) and GAPDH-R (5′-GCTAAGCAGTTGGTGGTGCA) for GAPDH. Predicted *C*_T_ (threshold cycle) values were exported into EXCEL worksheets for analysis. Comparative *C*_T_ methods were used to determine relative gene expression folds to β-actin, TBP or GAPDH, respectively.

### Plasmids

A plasmid-pGL3-147 that we constructed previously was used in this study [13]. It contains −133/+14 core promoter region of *GNMT* and a luciferase gene. Putative AREs were identified using MatInspector program (http://www.genomatix.de/). Based on the *in silico* screening, four putative AREs were identified: three AREs (ARE1-3) are located in the intron 1 of *GNMT* their locations were nucleotide numbers +266/+280, +379/+393 and +1114/+1128 of *GNMT* gene; another ARE (ARE4) is located in the intron 2 (+1788/+1802). The fifth putative ARE–ARE5 was identified by the investigator using a consensus sequence-nGnACnnnnnGTnCn deduced from those confirmed AREs published previously [15–18]. Both intron 1 and 2 fragments of the human *GNMT* gene were generated by PCR using the *GNMT* genomic DNA clone 6-1 [10] as a template. The primers used were PS6598 (5′-ATTACGCGTGTGCCCAGGCCGGG) and PA7834 (5′-ATTACGCGTCTGAGTACGGCCAGCGAG) for intron 1, and PS7963 (5′-GCGACGCGTGTATGCAGGTCTAGCCAG) and PA8385 (5′-GCGACGCGTCTAGGGGTCAGGAAGAGA) for intron 2. PCR products were digested with MluI and cloned into pGL3-147 to generate p147-Intron1 and p147-Intron2, respectively. The constructs containing wt (wild-type) or mutated ARE5 were generated by inserting the annealed SacI–NheI fragments to pGL3-promoter or pGL3-147. pGL3-promoter contains a SV40 (simian virus 40) promoter (Promega). Oligonucleotides were crA-SacI-F (5′-cTGGACAGCGTGTACCg) and crA-NheI-R (5′-ctagcGGTACACGCTGTCCAgagct) for wt ARE5, and mcrA-SacI-F (5′-cTAGGTAGCGTATCTCg) and mcrA-NheI-R (5′-ctagcGAGATACGCTACCTAgagct) for mutated ARE5. The pSG5-AR AR-expressing plasmid used in these experiments was provided by Dr Chawnshang Chang of the University of Rochester. The AR-responsive ARE-directed luciferase reporter plasmid (pMMTV(murine mammary tumour virus)-luc) was kindly provided by Dr Hsiu-Ming Shih of the Institute of Biomedical Sciences, Academia Sinica, Taiwan.

### RGA (reporter gene assay)

Cells from different cell lines mentioned above were seeded onto 12-well plates (1.2×10^5^ cells per well) and grown overnight in medium with 10% CS FBS for 24 h prior to transfection. The medium was refreshed 2 h prior to transfection. Transfection was performed via calcium phosphate co-precipitation. Cells were co-transfected with or without pSG5-AR and different luciferase gene constructs. Either pRL-TK or pSV40-β-Gal transfection was used for normalization of the transfection efficiency. The 16 h after transfection, cells were treated with solvent (ethanol) or 10 nM R1881 for 24 h and then they were harvested for reporter analysis. Bicalutamide was used as an androgen antagonist. Luciferase activity was measured using either Dual-luciferase reporter assay system or Luciferase Assay System (both from Promega); β-galactosidase activity was measured using a β-galactosidase Enzyme Assay System (Promega).

### EMSA (electrophoretic mobility shift assay)

Detailed procedures of EMSA have been described previously [13]. In brief, nuclear extracts were prepared from COS-1 cells. For AR expression, COS-1 cells were transfected with pSG5-AR using Lipofectamine™ reagent (Invitrogen) according to the manufacturer's protocols. Oligonucleotides were synthesized for wt and mt (mutated) GNMT putative AREs. Sequences were 5′-GGGTCTGTCTCAGCCTGTACTGCGCGGCCGCAGA-3′ for wt-ARE1, 5′-CCTGCCCGGCAGAACAGGCACTGCGAGTGCCCCGTG-3′ for wt-ARE2, 5′-CCTGTGCTCTAAACCACCATGTTCTACTGCCTTTTG-3′ for wt-ARE3/YY1, 5′-CCTGTGCTCTcAcCtcCCAgtggggACTGCCTTTTG-3′ for mt-ARE3/YY1, 5′-ATGGTGGACAGCGTGTACCGGAC-3′ for wt-ARE5, and 5′-ATGGTaGgtAGCGTaTctCGGAC-3′ for mt-ARE5 (putative ARE sequences are underlined; mutations are indicated by lowercase letters). Consensus ARE and YY1 oligonucleotides were used as a control, or for competition assay. Sequences were 5′-GTCTGGTACAGGGTGTTCTTTTT for conARE [19] and 5′-CGCTCCGCGGCCATCTTGGCGGCTGGT for conYY1 [20]. Oligonucleotides were annealed and labelled with γ-^32^P-ATP. Labelled probes were incubated with 4 μg nuclear extracts, 1 μg poly(dI-dC), and 1 μg BSA in 1 × binding buffer [10 mM HEPES, pH 7.9; 8% (v/v) glycerol; 0.05 mM EDTA; 2.5 mM MgCl_2_; 1 mM DTT (dithiothreitol); 0.05% (v/v) Triton X-100] for 30 min at room temperature (25°C). For our competition experiments, 50- and 100-folds of unlabelled double-stranded oligonucleotides were incubated for 15 min prior to adding the labelled probes. Antibodies to AR (sc-815) and YY1 transcription factor (sc-1703) were purchased from Santa Cruz Biotechnology. Complexes were separated on 4% (w/v) non-denaturing polyacrylamide gels, which were dried and analysed using autoradiography.

### ChIP (chromatin Immunoprecipitation) and PCR

LNCaP cells were cross-linked with 1% (v/v) formaldehyde for 20 min at room temperature and the chromatins were obtained through sonication. Immunoprecipitation was performed with anti-AR antibody (sc-816), anti-YY1 antibody (sc-1703) or normal rabbit IgG at 4°C overnight. Precipitates were washed and the samples were extracted with elution buffer and heated to 65°C to reverse the crosslink reaction. Subsequently, the DNA fragments were purified with phenol/chloroform. For ChIP assay with anti-AR, a pair of PCR primers was designed to span the putative AREs of GNMT exon 1: forward primer (5′-GGCTGCCAGCAGTGCTTATG); reverse primer (5′-CGATATACAGCTGCCACACG). PCR products were run on 2% (w/v) agarose gel and stained with ethidium bromide for visualization. For ChIP assay with anti-YY1, the immunoprecipitated DNA was analysed by real-time PCR as described above with two primer sets: p1 forward (5′-GGCGCGTGTCACCATGTCCC), p1 reverse (5′-CCCAGCGAAGGAAGGCATCA); p2 forward (5′-CGCCGTGTTAGCCAGGATGGT), p2 reverse (5′-GAGAGACAGACAAGCTGAGGCT).

## RESULTS

### The androgen agonist-R1881 induces GNMT expression in PCa cells

**Figure 1:**
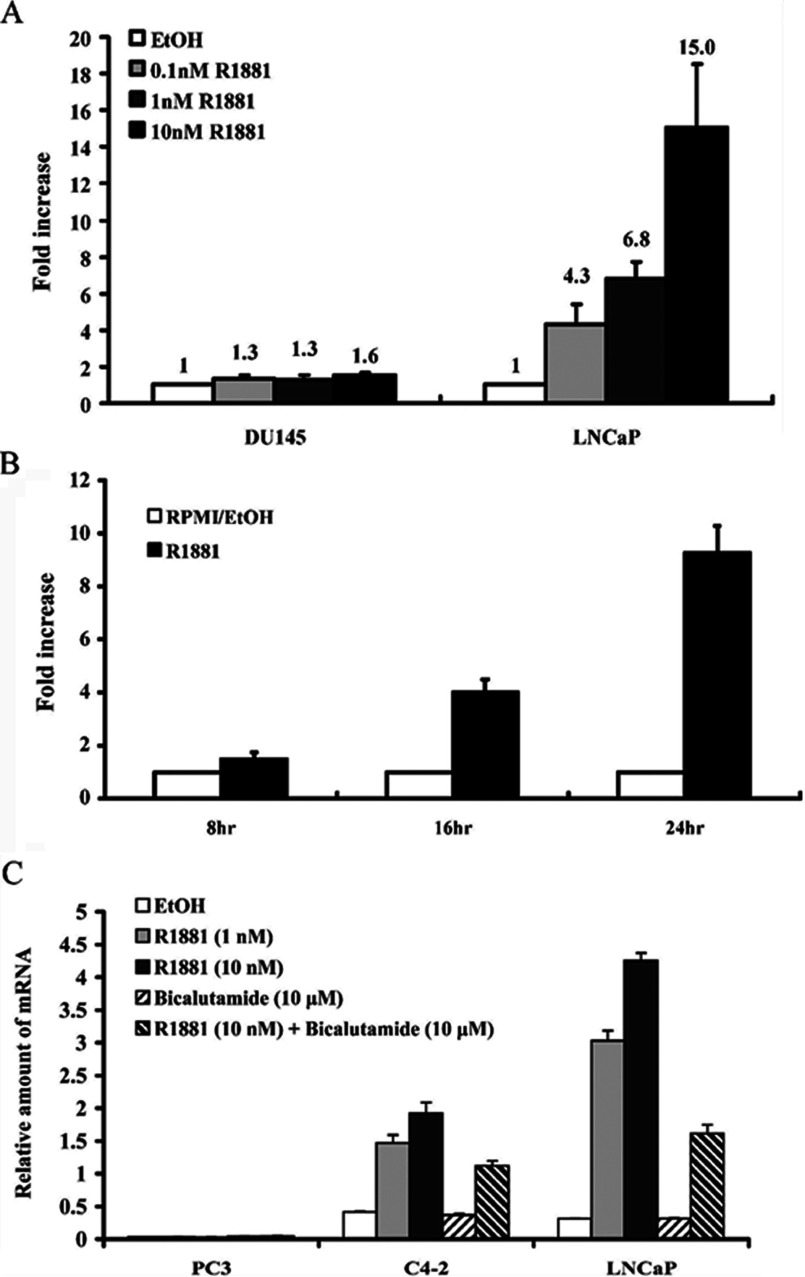
Androgen-induced GNMT expression in AR-expressing cells (**A**) LNCaP and DU145 cells were treated with R1881 at indicated concentrations for 24 h. (**B**) LNCaP cells were treated with 2 nM R1881 for indicated length of time. (**C**) PC3, C4-2 and LNCaP cells were treated with R1881 at indicated concentrations for 24 h. Bicalutamide (10 μM) was used as anti-androgens added in some condition. Quantitative real-time PCR was used to detect GNMT mRNA levels normalized to mRNA levels of β-actin (A), GAPDH (B) or TBP (C).

To investigate the relationship between androgen stimulation and GNMT expression, LNCaP cells that express AR were treated with R1881 for 24 h. Then, the GNMT mRNA expression levels were analysed using real-time PCR. The results showed that compared with the ethanol solvent control, GNMT mRNA expression levels in LNCaP cells were induced by R1881 in a dose-dependent manner (4.3-, 6.8- and 15-folds of induction for 0.1, 1 and 10 nM R1881 treatment, respectively) (Figure 1A). Time-course GNMT mRNA induction was also observed between 8 and 24 h after the cells were treated with 2 nM R1881 (Figure 1B). In contrast, negative results were obtained in AR-negative DU145 PCa cells (Figure 1A). In addition, GNMT mRNA expression levels in C4-2 cells were also induced by R1881 in a dose-dependent manner. The induction was suppressed by an androgen antagonist–bicalutamide in both LNCaP and C4-2 cells (Figure 1C). In consistent with previous finding, no effects on GNMT mRNA expression were observed in the other androgen-insensitive cell line-PC3 cells after R1881 treatment. Therefore GNMT can be induced by androgens in PCa cells expressing AR.

### Identification of the potential AREs in *GNMT*

**Figure 2:**
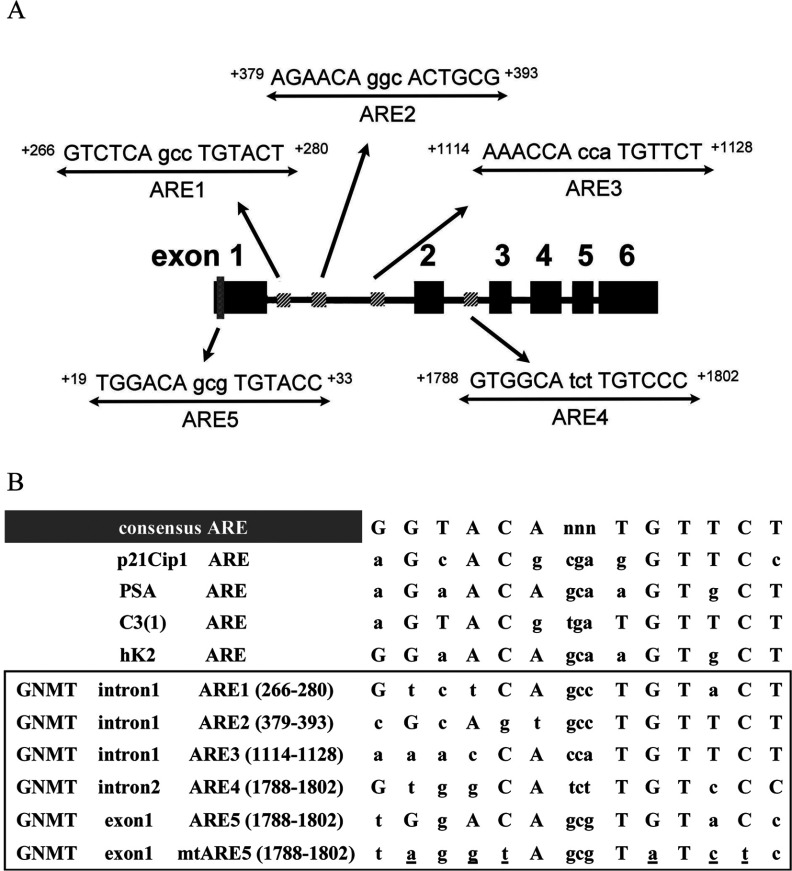
Schemes and sequences of ARE-like motifs (**A**) Potential ARE motifs within GNMT introns 1, 2 and exon 1 coding region are indicated. (**B**) Consensus comparisons of several well-established sequences and GNMT motifs for ARE1 to ARE5. Capital letters indicate matching residues with consensus sequences. Mutated ARE5 is listed and mutations are underlined.

**Figure 3:**
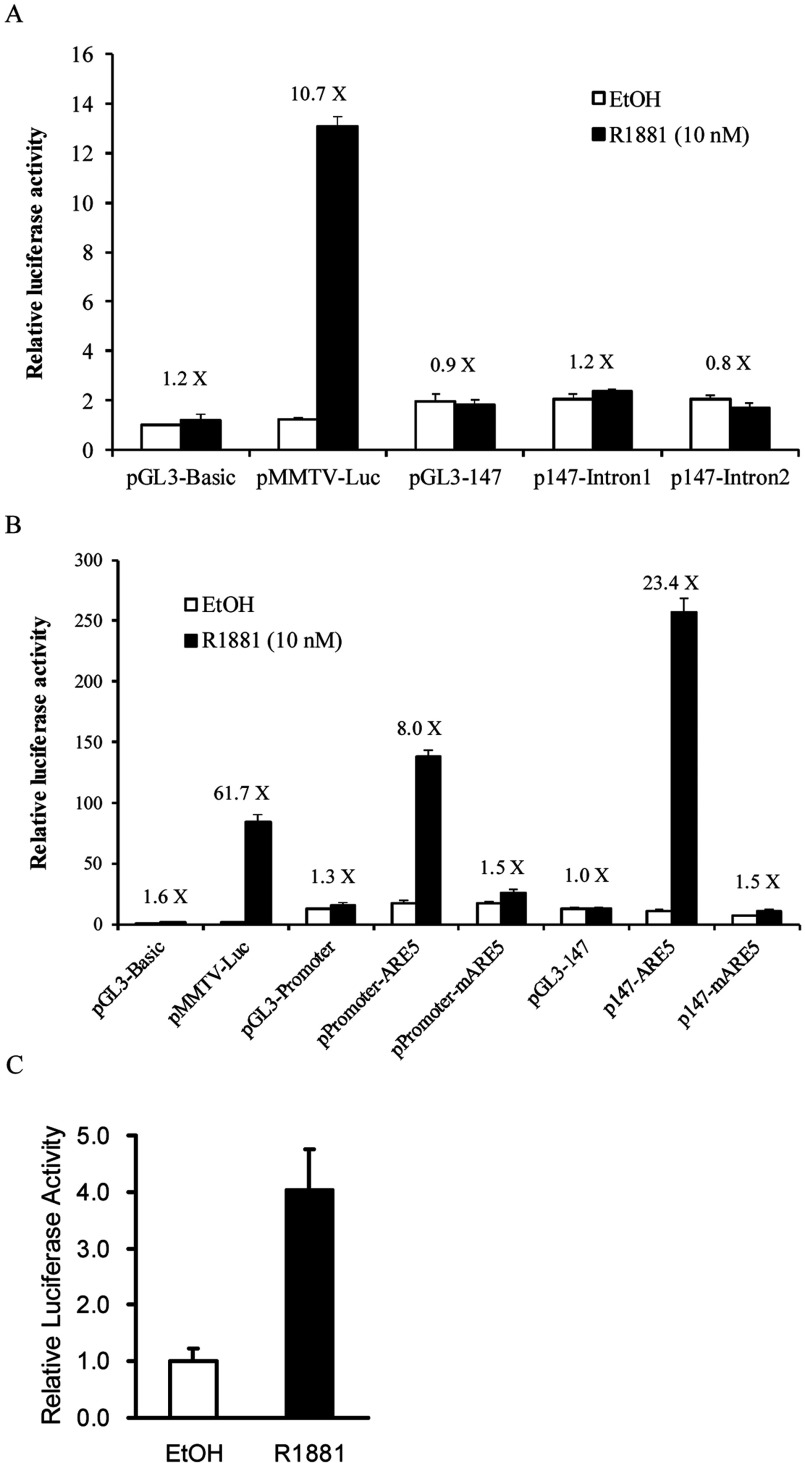
Transcriptional activity of GNMT intron 1, 2 and +19/+33 fragments as assessed by RGAs (**A**) HuH-7 cells were co-transfected with or without pSG5-AR and different luciferase gene constructs with GNMT intron 1 or intron 2. pSG5-AR plasmids were used to express ARs. pRL-TK transfections were used for normalizing transfection efficiency. (**B**) HuH-7 cells were co-transfected with pSG5-AR and different luciferase gene constructs with GNMT ARE5 or mutant ARE5. Cells were treated with solvent (0.1% (v/v) ethanol) or 10 nM R1881 for 24 h prior to harvesting for reporter analyses. (**C**) LNCaP cells were co-transfected with pRL-TK and p147-ARE5. Cells were treated with solvent (0.1% ethanol) or 10 nM R1881 for 24 h prior to harvesting for reporter analyses.

To determine whether GNMT is regulated by AR directly, we tried to identify its responsive elements using *in silico* analyses with MatInspector program. The nucleotide sequences subject to the analysis spanning about 6.4 kb upstream and 3.7 kb downstream of the transcriptional start site of *GNMT*. The results showed that there are three putative AREs located in the intron 1 of *GNMT*: ARE1 (GTCTCAgccTGTACT) at +266/+280; ARE2 (AGAACAggcACTGCG) at +379/+393; ARE3 (AAACCAccaTGTTCT) at +1114/+1128. In addition, another ARE was found in the intron 2: ARE4 (GTGGCAtctTGTCCC) at +1788/+1802 (Figure 2). Subsequently, we used RGAs to test whether those putative GNMT AREs are really functioning. Two plasmids-p147-intron1 and p147-intron2, which contain either intron 1 or intron 2 sequences at the upstream region of the GNMT core promoter (−133/+14) and luciferase gene were constructed. Subsequently, p147-intron1 or p147-intron2 was used to cotransfected HuH-7 cells with an AR-expressing plasmid-pSG5-AR. After 16 h of transfection, cells were treated with solvent (ethanol) or R1881 for 24 h and then they were harvested for reporter analysis. We did not observe any significant increase of luciferase enzyme activity in HuH-7 cells transfected with either p147-intron1 or p147-intron2 (Figure 3A).

Since none of the putative AREs identified by *in silico* analysis were truly functioning, we decided to locate the AREs using the consensus sequence (nGnACnnnnnGTnCn) deduced from those AREs published previously. A putative ARE (ARE5, TGGACAgcgTGTACC at +19/+33) was located in the exon 1 coding region of *GNMT* (Figure 2). To confirm the functionality of ARE5, we subcloned the ARE5 sequence into pGL3-Promoter and pGL3-147 and the resultants plasmids-promoter-ARE5 and p147-ARE5 were used in the RGA. The results showed that compared with the solvent control, R1881 treatment induced approximately 8-fold and 23-fold, respectively, in cells transfected with promoter-ARE5 and p147-ARE5, respectively (Figure 3B). In addition, no induction was observed in cells transfected with plasmid containing mutant ARE5 (Figure 3B). Furthermore, we confirmed that endogenous AR can mediate R1881 induced reporter activity by transfecting p147-ARE5 into LNCaP cells (Figure 3C). Therefore ARE5 (+19/+33) is the potential ARE in *GNMT*.

### Identification of a YY1-binding motif in the intron 1 of GNMT at +1118/+1126

**Figure 4:**
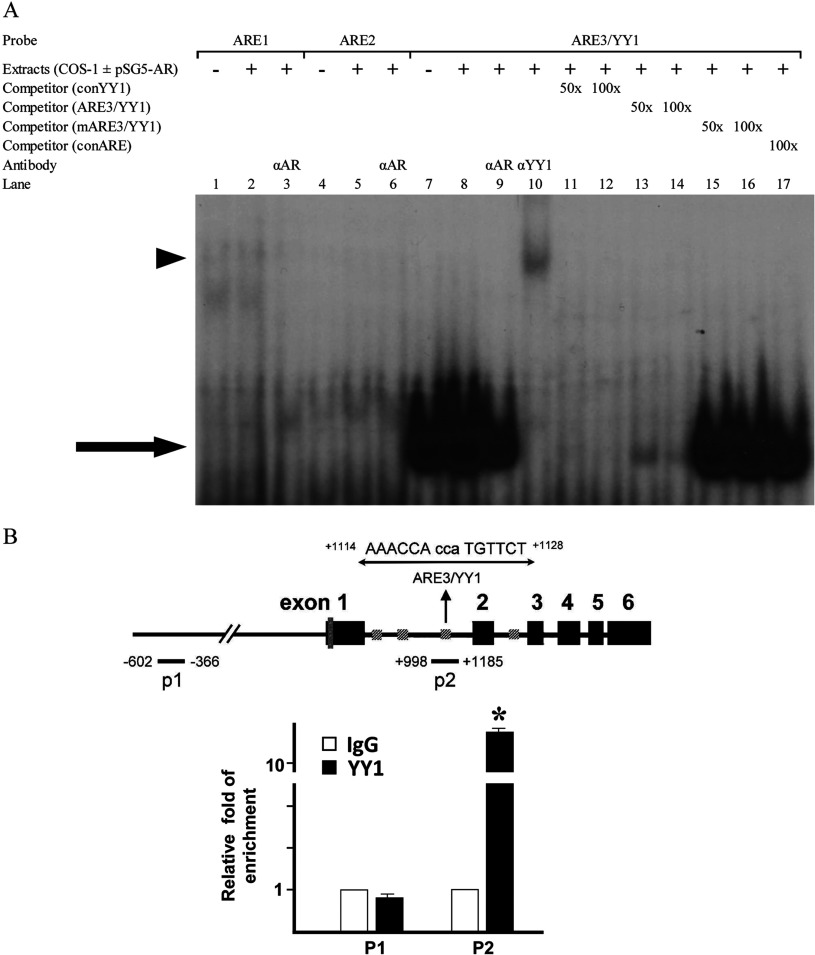
Binding properties of a potential ARE in GNMT exon 1 coding region (**A**) EMSAs were performed in the presence of AR containing COS-1 cell nuclear extracts (pSG5-AR +) or extracts from cells transfected with empty vectors (pSG5-AR -). Fifty- or one-hundred-fold quantities of cold oligos were used in the competition assay; and rabbit polyclonal antibody anti-YY1 was used in the supershift assay. Unlike oligos representing ARE3/YY1 sequences, oligos containing a potential ARE1 or a potential ARE2 did not specifically bind to nuclear proteins. The competition assay (lanes 11–17) and the supershift assay (lanes 9 and 10) demonstrated that the ARE3/YY1 sequences bound to YY1, not AR. Arrow, YY1-DNA complex; arrowhead, YY1 supershifted band. (**B**) Schematic of the positions of PCR amplicons on *GNMT* used for the ChIP assay is shown at Top. ChIP analysis was performed to determine the interaction between YY1 and *GNMT* gene in LNCaP cells. The YY1-binding were found to be enriched in the p2 region, but not in p1 region. Error bars represent the means±S.D. determination.*P<0.001. The graph represents data from three independent experiments.

We performed EMSA to evaluate the binding properties of those putative AREs of *GNMT*. Complementary oligonucleotide probes were synthesized for wt and mutated putative AREs in intron 1 of *GNMT*. Labelled oligonucleotides were incubated with nuclear extracts from COS-1 cells transfected with pSG5-AR and used to determine their capabilities to bind to nuclear proteins. The results showed that among oligonucleotides containing ARE1, ARE2 or ARE3, nuclear proteins only bind to the oligonucleotide containing ARE3 (Figure 4A). Since anti-AR antibody did not cause supershift in the EMSA (Figure 4A, lane 9), we suspected that the nuclear protein binds to the oligonucleotide containing ARE3 was not AR. We re-examined the sequence and found a putative YY1 binding site (CaCCATGTT, +1118/+1126) overlaps with ARE3. Subsequently, we performed EMSA and found that anti-YY1 antibody caused a supershift (Figure 4A, lane 10). In addition, oligonucleotide containing consensus YY1 sequence had positive results with dose-responsive manner in the competition assay (Figure 4A, lanes 11 and 12). In contrast, no competition was found in the EMSA using oligonucleotides containing either mutant ARE3/YY1 sequence or consensus ARE (Figure 4A, lanes 15–17). In order to further characterize the binding properties of ARE3/YY1 sequence, we performed ChIP assays in LNCap cells. Two representative region of the GNMT gene were investigated. As shown in Figure 4(B), YY1 was found to be enriched in the p2 region (+998 to +1185), containing the YY1 binding site (+1118/+1126) in the intron 1 of *GNMT* gene in LNCaP cells. Taken together, these results demonstrate a YY1-binding motif located in the intron 1 of *GNMT* gene.

### Confirmation of the ARE5 of *GNMT* gene by using EMSA

**Figure 5:**
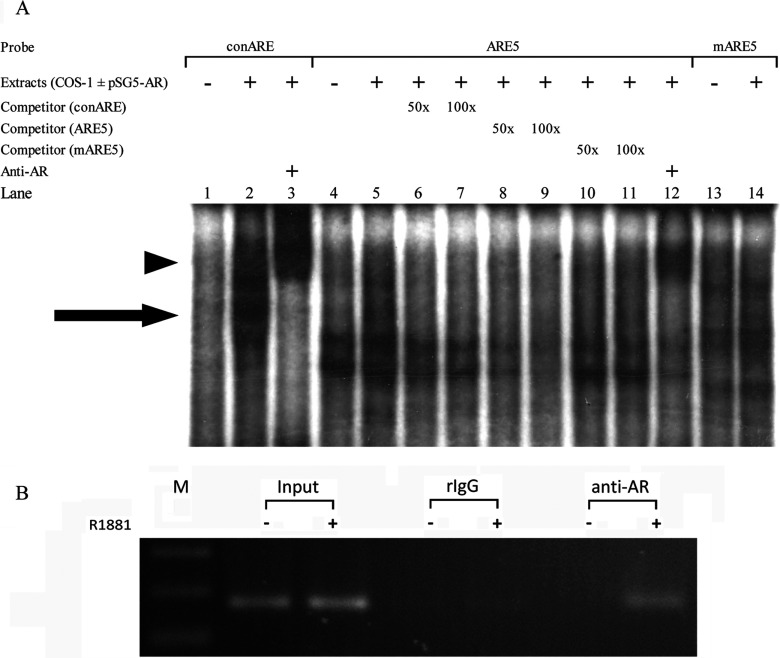
Androgen promotes interaction between AR and ARE5 in GNMT exon 1 coding region (**A**) A consensus ARE binding probe (lanes 1–3) and ARE5 in the human GNMT exon 1 coding region (lanes 4–12) and mutant (lanes 13,14) were incubated with nuclear extracts prepared from COS-1 cells transfected without (lanes 1, 4 and 13) or with pSG5-AR (all other lanes) in the presence of no competitor; of 50- or 100-fold molar excess of consensus, wt, or mutant cold oligonucleotide as a competitor; and rabbit polyclonal antibody anti-AR was used in the supershift assay. Arrow, AR–DNA complex; arrowhead, AR supershifted band. (**B**) LNCaP cells were treated with 10 nM R1881 or 0.1% ethanol for 24 h. AR binding to GNMT exon 1 ARE5 was determined by ChIP assays. Immunoprecipitated DNA (by anti-AR antibody or rabbit IgG) was purified, and regions containing ARE sites were amplified by PCR. A positive signal was obtained after R1881 treatment and ChIP with anti-AR antibodies, followed by PCR amplification using the indicated primers.

As showed in Figure 5(A), the consensus ARE probe was used as a control to demonstrate the positions of the AR–ARE and anti-AR antibody–AR–ARE complexes in the EMSA. The results show that the wt ARE5 probe was capable of binding the AR present in the nuclear extracts of COS-1 cells (Figure 5A, lane 5) and the complex was diminished when excess cold competitors-consensus ARE (Figure 5A, lanes 6 and 7) or ARE5 (Figure 5A, lanes 8 and 9) were added to the reaction. However, the complex was not diminished when mutant ARE5 was added to the reaction (Figure 5A, lanes 10 and 11). Furthermore, a supershift band was observed when anti-AR antibody was added to the reaction (Figure 5A, lane 12). These results indicate that the ARE5 in the exon 1 coding region of *GNMT* interacts with AR directly.

### AR binds to ARE5 in response to R1881 treatment *in vivo*

Finally, we employed ChIP assay with PCR to confirm the interaction between AR and ARE5 *in vivo*. Chromatin fragments were immunoprecipitated by anti-AR antibodies from LNCaP cells which had been treated with R1881 for 4 h. DNA from immunoprecipitated complexes were isolated and amplified by PCR with primers for ARE5 region (+19/+33 in GNMT exon 1). As shown in Figure 5(B), a strong signal was observed in R1881-treated cells, while no signal was detected in the cells without R1881 treatment. It indicates that androgen agonist R1881 promotes the interaction between AR and the ARE5 of *GNMT* gene.

## DISCUSSION

In this study, we found that androgen is capable of inducing promoter activity of *GNMT* through the AR signalling pathway. Our data showed that the mRNA level of GNMT in LNCaP PCa cells was induced by androgen agonist-R1881 in a time- and concentration-dependent manner (Figure 1). Although we showed that R1881 can induce GNMT expression at early as 8 h post-treatment, the kinetics of *GNMT* induction by AR needs further investigation. Nonetheless, our finding suggests *GNMT* is a target gene of AR.

Human cancer cells often display abnormal DNA methylation patterns [21]. Aberrant hypermethylation of the promoter region can result in the silencing of tumour suppressor genes; several tumours (including PCas) display genome-wide hypomethylation that promotes chromosomal instability [22,23]. SAM is a naturally occurring compound whose roles in many biochemical processes are well documented. The SAM methyl group can be transferred to numerous substrates (e.g., DNA, histone and glycine), and SAM can be converted to SAH, a potent inhibitor of many SAM-dependent methyltransferases except GNMT [24]. Therefore GNMT has been considered to play an important role in maintaining a constant SAM/SAH ratio by transferring methyl groups to glycine to form sarcosine [25]. Since androgen agonist can induce *GNMT* gene expression, androgen-induced GNMT expression may affect DNA methylation and chromosome stability, thereby contributing to PCa tumorigenesis.

According to the results of EMSA and ChIP assay, AR binds directly to ARE5 located at +19/+33 of exon 1 (Figures 5). Previously, Sreekumar et al. used ChIP-sequencing assay to show that the AR-binding site of *GNMT* is located in the promoter region flanking the transcription start site [12]. In this study, we used a −1812/+14 luciferase reporter construct that contains the site described by Sreekumar et al. to evaluate AR-mediated induction of GNMT gene expression. However, we failed to confirm their results. We did not find significant increase of luciferase activity after R1881 treatment (results not shown). Instead, our reporter assay, EMSA and ChIP data indicate the ARE is located at +19/+33 of exon 1 coding region.

In addition to acting via intracellular AR-mediated pathway, androgens can also bypass AR-associated action and induce conventional second messenger signal transduction cascades that are considered non-genomic [26]. Further studies needed to elucidate this non-genomic effect of androgens and its relationship with GNMT-related pathogenesis of cancer.

It is important to note that in this study, we found a motif at +1118/+1126 in the intron 1 of *GNMT* can bind YY1 both *in vitro* and *in vivo* (Figure 4). Overexpression of YY1 has been detected by using immunohistochemical staining in a large series of PCa and prostatic intraepithelial neoplasia tissues [27]. In addition, microarray analysis revealed that YY1 is up-regulated in HCC [28]. Since the motif that we identified for the YY1 binding is associated with transcriptional repression [29], we speculate that overexpression of YY1 may be involved in the down-regulation of *GNMT* in HCC and PCa. It has been reported that YY1 can co-regulate transcriptional activity with NF-Y (nuclear factor-Y) via separate YY1 and NF-Y binding sites [30]. Alternatively, YY1 can interact with NF-Y to enhance promoter activity [31]. In addition, YY1 also regulates the transcriptional activity of AR [32]. Therefore YY1 maybe play an important role in regulating *GNMT* expression in a regular or AR-induced manner.

It has been reported that activation of the PI3K (phosphoinositide 3-kinase)-PKB (protein kinase B (also called Akt) pathway promotes AR ubiquitylation and leads to AR degradation via a proteasome-dependent pathway [33]. Previously, we identified DEPDC6/DEPTOR [(DEP domain-containing mTOR (mammalian target of rapamycin)-interacting protein)] as a novel GNMT-interacting protein [34]. Peterson et al. identified that DEPTOR interacts with mTOR directly and serves as an mTOR inhibitor. Overexpression of DEPTOR activates Akt via the inhibition of a negative feedback loop from S6K (S6 kinase) to PI3K [35]. We showed that GNMT can neutralize DEPTOR's effect on Akt activation [34]. Therefore there might be a feedback regulation between androgen and GNMT. Further study is needed to elucidate the consequences of such complicated interaction in the pathogenesis of PCa and HCC.

GNMT is a susceptibility gene for both HCC and prostatic cancer [36,11]. It has been reported that liver tumour tissues have significantly higher levels of AR than tumour-adjacent tissues [37–39]. As a major risk factor associated with HCC in males, HBV (hepatitis B virus) has been attributed to elevated androgen levels and enhanced AR-mediated activity in the host. Chiu et al. found that HBV X protein enhances AR-responsive gene expression in an androgen level-dependent manner [40]. Our present results suggest that *GNMT* is a direct AR target gene in PCa, we suspect that AR signalling may also play an important role in GNMT regulation in liver cancer. Vitamin A and its derivatives are known to induce hepatic GNMT and DNA hypomethylation in rats [41,42]. Recently, we found that HBV can activate GNMT gene expression (unpublished results). Therefore HBV and androgens may induce GNMT expression cooperatively and trigger cellular senescence and liver regeneration, which initiate the tumorigenesis of HCC. Further research is required to clarify the mechanisms involved.

In conclusion, we found that GNMT is an AR-targeted gene with its functional ARE located at +19/+33 of the first exon in this study. These results are valuable for the study of the influence of androgen on the gene expression of GNMT especially in the pathogenesis of PCa and HCC.
